# Potential Role of Gene Regulator NFAT5 in the Pathogenesis of Diabetes Mellitus

**DOI:** 10.1155/2020/6927429

**Published:** 2020-09-15

**Authors:** Lusha Cen, Fengling Xing, Liying Xu, Yi Cao

**Affiliations:** ^1^Department of Ophthalmology, The First Affiliated Hospital of Zhejiang Chinese Medical University, Hangzhou, China; ^2^Department of Dermatology, Hangzhou Hospital of Traditional Chinese Medicine, Hangzhou, China; ^3^Department of Emergency, The First Affiliated Hospital of Zhejiang Chinese Medical University, Hangzhou, China; ^4^Department of Dermatology, The First Affiliated Hospital of Zhejiang Chinese Medical University, Youdian Rd. 54th, Hangzhou 310006, China

## Abstract

Nuclear factor of activated T cells 5 (NFAT5), a Rel/nuclear factor- (NF-) *κ*B family member, is the only known gene regulator of the mammalian adaptive response to osmotic stress. Exposure to elevated glucose increases the expression and nuclear translocation of NFAT5, as well as NFAT5-driven transcriptional activity in vivo and in vitro. Increased expression of NFAT5 is closely correlated with the progression of diabetes in patients. The distinct structure of NFAT5 governs its physiological and pathogenic roles, indicating its opposing functions. The ability of NFAT5 to maintain cell homeostasis and proliferation is impaired in patients with diabetes. NFAT5 promotes the formation of aldose reductase, pathogenesis of diabetic vascular complications, and insulin resistance. Additionally, NFAT5 activates inflammation at a very early stage of diabetes and induces persistent inflammation. Recent studies revealed that NFAT5 is an effective therapeutic target for diabetes. Here, we describe the current knowledge about NFAT5 and its relationship with diabetes, focusing on its diverse regulatory functions, and highlight the importance of this protein as a potential therapeutic target in patients with diabetes.

## 1. Introduction

Diabetes mellitus is a serious, long-term disease characterized by increased blood glucose levels. It is also the top 10 highest fatality rate disease such as heart disease, cancer, and chronic lower respiratory disease [1]. According to the International Diabetes Federation Diabetes Atlas (9^th^ edition), the global diabetes prevalence in 2019 was estimated to be 9.3% (463 million people) and is expected to increase to 10.2% (578 million) by 2030 and 10.9% (700 million) at 2045 [2]. Diabetes is a complex disease involving various factors. Although blood glucose control is currently the main treatment for diabetes, numerous serious and life-threatening complications can occur, leading to an increased need for medical care, reduced quality of life, and stress on patients and their families. The number of deaths resulting from diabetes and its complications in 2019 was estimated to be 4.2 million [2]. The effectiveness of other treatments for diabetes remains limited. Thus, new therapy targets for diabetes are urgently needed.

Nuclear factor of activated T cells 5 (NFAT5), identified in 1999 as a member of the Rel/nuclear factor- (NF-) *κ*B family, is the only known gene regulator of the mammalian adaptive response to osmotic stress [3]. Hyperglycemia is a predominant diagnostic criterion for diabetes. Exposure to elevated glucose increases NFAT5 expression and nuclear translocation, as well as NFAT5-driven transcriptional activity in mammals [4]. NFAT5 is ubiquitously expressed in all tissues and cell types. Recent data indicates the profound biological importance of the mammalian osmotic stress response in view of NFAT5-dependent gene regulation in tissues that are often subjected to osmotic stress, such as the kidney, brain, vascular, and eye [3, 5]. However, once having anisotonic disorders, i.e., diabetes, the pathogenic response of NFAT5 could result in diabetes-related complications, such as diabetic nephropathy, diabetic retinopathy, and atherosclerosis. Single-nucleotide polymorphisms in the introns of NFAT5 are shown to be correlated with diabetes risk in various human cohorts (Table 1) [6–10], and elevated expression of NFAT5 is closely associated with diabetes progression [11]. Although there is glycemic control in an isotonic environment, the progression of diabetic complications is still ongoing. NFAT5 can be activated in the absence of hyperosmotic stress, including activation of the Toll-like receptors and inflammatory cytokines, and plays a pathogenic role [12]. NFAT5 may also have unknown and widely distributed effects rather than an osmotic protective role singly in diabetes.

In this review, we introduce the structure and diverse functions of NFAT5 and its potential as a therapeutic target in diabetes.

## 2. Structure of NFAT5

**Figure 1:**
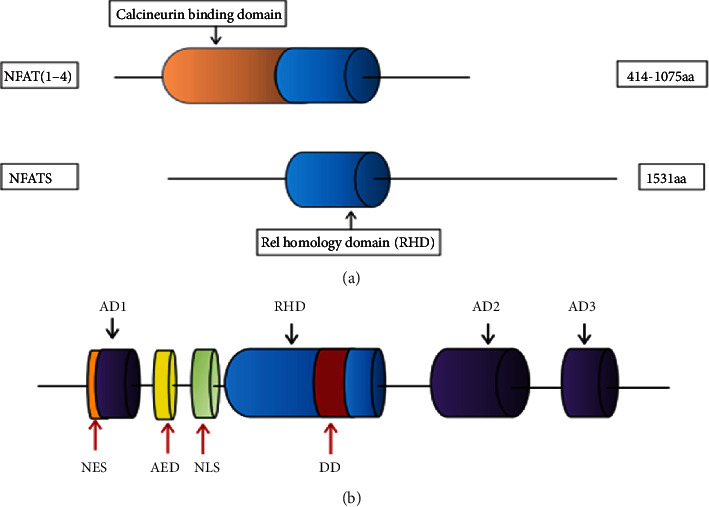
Structure of nuclear factor of activated T cells 5 (NFAT) family members. (a) Comparison of structures of NFAT5 and NFAT1-4. Rel homology domain (RHD) exists in NFAT1-5. NFAT5 lacks a calcineurin-binding domain. (b) Specific composition of NFAT5: three transactivation domains (AD): AD1, AD2, and AD3; nuclear export sequence (NES); auxiliary export domain (AED); nuclear localization signal (NLS); dimerization domain (DD).

NFAT5 is a member of the Rel/NF-*κ*B protein family. Its DNA-binding domain shows sequence homology with the Rel homology domain (RHD) and is highly similar to those of other NFAT members (NFAT1-4). In contrast to conventional NFAT, NFAT5 lacks a calcineurin-binding domain outside of the DNA-binding domain and shares few amino acids with NF-*κ*B [13, 14]. The Drosophila genome encodes a single NFAT-like protein that is closely related to NFAT5, suggesting that NFAT5 diverged from Rel proteins early during evolution [15]. The NFAT5 protein contains a leucine-rich canonical nuclear export sequence (NES) located in the first amino acids, N-terminal serine/threonine and proline-rich region (transactivation domain 1; AD1), auxiliary export domain (AED), consensus bipartite nuclear localization signal (NLS), dimerization domain (DD) within the RHD, and C-terminal low-complexity region (glutamine and serine/threonine-rich region, AD2, and AD3) [12, 16, 17] (Figure 1). These distinct structural features of NFAT5 suggest that NFAT5 is involved in additional signaling pathways that have not been determined.

NFAT5 protein exists in an active equilibrium state both in the cytoplasm and nucleus under isotonic conditions. An early work showed activated NFAT5 is in a constitutively dimeric form with the ability for DNA binding and transcription, similar to NF-*κ*B, without cooperation with FOS or JUN and calcineurin activation [5, 14]. FOS and JUN are intermediary transcription factors that form heterodimers containing leucine-zipper and basic region domains and induce interactions among transcription factors. These molecules bind to different sequence elements and require the bending of intervening DNA and juxtaposition of interacting molecular surfaces in the appropriate orientation [18]. A later work showed that FOS and JUN coimmunoprecipitate with NFAT5, indicating physical association. In addition, small interfering RNA (siRNA) knockdown of either FOS or JUN inhibits high NaCl-induced increase of mRNA abundance of the NFAT5 target genes [19]. It is possible that NFAT5 might be capable of interacting with FOS and JUN proteins through contact residues and mechanisms different from those used by the calcineurin-activated NFATc, although its structural basis and functional relevance are still being explored.

NFAT5 is regulated under hypertonic stress at multiple levels. Within 30 min of a cell becoming hypertonic, NFAT5 is phosphorylated by kinases and translocates into the nucleus. The nuclear distribution and abundance of NFAT5 increase in hours following this translocation [16, 20]. Numerous kinases have been shown to be involved in regulating both cytosol-to-nuclear translocation and the functions of transcriptional activation domains within the protein. Some kinases, such as p38*α*, Fyn, ataxia telangiectasia mutated (ATM), and protein kinase A (PKA), play a promoting role in the activation of NFAT5, whereas others, such as glycogen synthase kinase 3*β* (GSK-3*β*), have inhibitory effects on NFAT5 (Table 2). NFAT5 is the only transcription factor known to undergo bidirectional nucleocytoplasmic trafficking in response to hypertonicity and hypotonicity [16]. Nuclear import of NFAT5 is mediated by the NLS under hypertonic conditions, whereas its nuclear export is mediated by NES and AED under isotonic conditions. NFAT5, similar to many other nucleus-targeting proteins, contains two consensus clusters of basic amino acids. Only the first basic cluster contributes to nuclear import, and therefore, the NLS is monopartite [16].

## 3. Physiological Role of NFAT5

**Figure 2:**
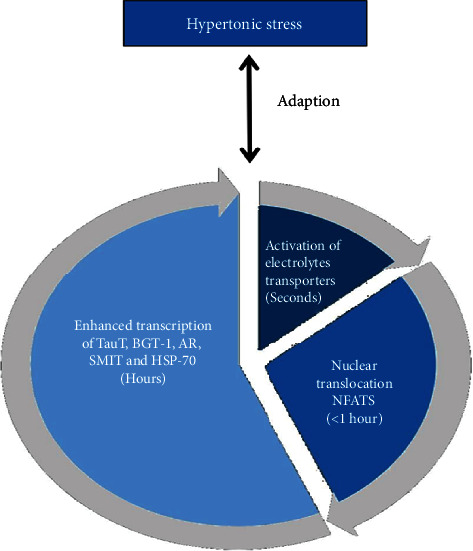
Cell adaption to hypertonic stress. Nuclear factor of activated T cells (NFAT) 5, taurine transporter (TauT), betaine GABA transporter (BGT)-1, aldose reductase (AR), sodium/myoinositol transporter (SMIT), and heat shock protein- (HSP-) 70.

Subsequent to the hypertonic challenge, there is a net efflux of water from the cell accompanied by cell shrinkage. Hypertonic stress activates electrolyte transporters, including the Na^+^-K^+^-Cl^−^ cotransporter, Na^+^/H^+^ exchanger, and Cl^−^/HCO_3_^−^ exchanger, within a few seconds to equalize the difference in ion concentrations between the extracellular and intracellular compartments and restore the cell volume [35]. However, this rapid response increases intracellular ionic strength and has various deleterious effects on cell functions, such as the denaturation of proteins and DNA [36]. To restore biochemical homeostasis under hypertonic stress, mammalian cells elicit relatively slow genetic osmoadaptive responses over several hours [37]. Extracellular hypertonic stress rapidly induces nuclear import of NFAT5 via the NLS and then enhances the transcription activity of NFAT5 target genes by binding to tonicity enhancer elements in regulatory regions including the taurine transporter (TauT), betaine GABA transporter 1 (BGT-1), aldose reductase (AR), sodium/myoinositol transporter (SMIT), and heat shock protein- (HSP-) 70 [37, 38]. TauT, BGT-1, and SMIT transport taurine, betaine, and myoinositol, respectively, into cells, and AR catalyzes the conversion of glucose to sorbitol. Accumulation of compatible osmolytes of taurine, betaine, myoinositol, and sorbitol results in the gradual replacement of intracellular electrolytes and contributes to the substitution of their osmotic activity to return to a near-normal osmotic pressure without perturbing macromolecular structure and function. HSP-70 promotes cell survival and inhibits apoptosis under hyperosmotic conditions [37] (Figure 2).

NFAT5 dimerization regulates the gene transcription of cytokines in response to osmotic stress, such as lymphotoxin- (LT-) *β*, interleukin- (IL-) 6, tumor necrosis factor- (TNF-) *α*, and monocyte chemoattractant protein- (MCP-) 1 [14, 39, 40]. NFAT5 participates in specific aspects of the expression of genes involved in host defense other than osmotic protective genes in lymphocytes. NFAT5 is essential for inducing the key antimicrobial gene inducible nitric oxide synthase (iNOS) in response to low and high doses of Toll-like receptor (TLR) agonists. In vivo, NFAT5 is necessary for effective immunity against Leishmania major, a parasite whose clearance requires TLRs and iNOS expression in macrophages [39]. Enhanced immune responses under these conditions improve cell survival.

NFAT5 is an essential gene regulator for tissues or organs with a high hyperosmotic pressure risk, such as the kidney, eye, skin, cardiac tissue, skeletal muscle, and brain. NFAT5 from upstream regulates aquaporin (AQP) 1, AQP2, urea transporter (UT-A), and AQP4 [41–45]. AQP1 is involved in water reabsorption from the lumen of the thin descending limb, and it is in the collecting ducts of the kidney [41]. AQP2 regulates water permeability in a renal system [42–44]. UT-A plays a critical role that affects urea accumulation in the renal medullary interstitium [44]. AQP4 maintains water homeostasis in astrocytes [45]. Apart from that, NFAT5 also protects against ischemic damage [46–48], viral or bacterial infection [39, 49], UV stimuli [50], and drug toxicity injury [51] as well as maintains mammal's physiological functions [52–54].

Collectively, studies have suggested that the combined effects of these downstream targets contribute to the cytoprotective functions of NFAT5.

## 4. Contradictory Roles of NFAT5 in Diabetes

NFAT5 plays a protective role under normal conditions. As the course of diabetes progresses, the physiological function of NFAT5 is altered. The response of NFAT5 to hyperosmotic stress may become a pathogenic factor that aggravates disease. Distinguishing between the physiological and pathological effects of NFAT5 remains difficult. Diabetic complications are closely correlated with organs in which NFAT5 is highly expressed. Thus, NFAT5 plays a protective role under normal conditions and a destructive role under pathological conditions.

### 4.1. Overwhelmed Physiological Role of NFAT5

Thresholds of osmotic pressure and time limits exist for NFAT5 to be able to maintain normal physiological functions. Cells from different organs present different thresholds of osmotic pressure. Cells from the renal medulla are physiologically exposed to a hypertonic environment with osmolality values of approximately 1000 mOsm [55]. In contrast, primary cells such as human limbal epithelial cells exposed to 305-500 mOsm [56], myoblasts to 280-443 mOsm [57], human aortic endothelial cells to 285–460 mOsm [58], and human keratinocytes to 450–600 mOsm [59] are not physiologically exposed to a hypertonic environment and show a lower threshold (<600 mOsm) for NFAT5 activation. Exposure to moderately elevated glucose (26–51 mOsm/L) can increase the expression and nuclear translocation of NFAT5 [4]. Acute exposure to extremely elevated glucose may disrupt the physiological functions of cells or tissues. Five-day-old cultured fibers with elevated extracellular glucose (50 mmol/L) after 24–48 h alter Ca^2+^ signals. Skeletal muscle fibers from diabetic mice showed elevated levels of NFAT5 protein expression and disrupted transverse tubular system morphology but normal Ca^2+^ homeostasis. Fibers from control mice experimentally exposed to elevated glucose exhibited altered Ca^2+^ signals [4]. These results demonstrate that NFAT5 slowly adapts to elevated hyperosmotic stress and is sensitive to moderate increases in extracellular osmolality. Overwhelmed adaption of NFAT5 is likely to occur during hyperglycemic crisis in patients with uncontrolled diabetes, particularly during a hyperglycemic hyperosmolar state (HHS), a life-threatening acute metabolic complication. Plasma glucose level > 33.3 mmol/L and increased serum osmolality > 320 mOsm/L are typically observed in HHS [60]. HHS often develops over days to weeks, with symptoms observed over several days; this complication has a mortality rate as high as 20% [59]. Rapid elevation of osmotic pressure affects the adaption of NFAT5 under HHS. Controlling NFAT5 function is critical for the treatment of HHS.

The expression of NFAT5 is not maintained or increased over time. Under hyperosmotic conditions for 3 h, both elevated production and translocation of NFAT5 from the cytosol to the nucleus were observed in human limbal epithelial cells [56]. In myoblasts, NFAT5 mRNA expression was increased after 7 h of exposure to an osmolarity of 443 mOsm/L, with a subsequent significant increase in the NFAT5 protein level [57]. The transcription and expression of NFAT5 decreased after reaching a peak between 24 and 48 h under hyperosmotic stress [56–58]. The expression of NFAT5 may decrease under long-term hyperosmotic stress. The expression levels of osmotic protective targets of NFAT5 including taurine [61, 62], betaine [63], and inositol [64] are lower in patients with diabetes than in healthy people. NFAT5 regulates renal urinary concentrations. Increased urinary excretion of betaine [63] and inositol [64, 65] has been observed in patients with diabetes. Further studies are needed to determine whether these effects are related to NFAT5. Previous research demonstrated that the absence of NFAT5 leads to cell proliferative disorder, T cell death, cataract, cardiac failure, kidney failure, or even embryonic death [53, 54, 66]. The protective adaptation of NFAT5 cannot maintain normal functions in patients with diabetes. Thus, in diabetes therapy, NFAT5 target osmolytes can be supplemented. Betaine administration was shown to improve glucose homeostasis in mice [67]. A randomized, double-blinded, placebo-controlled trial showed that people with obesity and prediabetes administered with betaine orally for 12 weeks exhibited reduced fasting glucose levels [68]. Taurine ameliorated the complications of liver injury diabetic rats [69]. Taurine supplementation improved some oxidative stress indices and inflammatory biomarkers in patients with type 2 diabetes mellitus (T2DM) [70]. Inositol is an emerging novel therapy for treating gestational diabetes mellitus. Evidence from four clinical trials of antenatal dietary supplementation with myoinositol during pregnancy involving 567 women who were 11–24-week pregnant shows a potential benefit for reducing the incidence of gestational diabetes [71].

Hyperosmotic stress functions as a potent inflammatory stimulus to release proinflammatory cytokines that prevent cell apoptosis. When exposed to the proinflammatory cytokine interferon- (IFN-) *γ* with IL-1*β*, NFAT5 mainly colocalizes with histone deacetylase 6 in the cytoplasm of unaffected myoblasts and is not translocated to the nucleus; NFAT5 mRNA and protein levels are not increased [57]. Many patients with diabetes suffer from muscular disorders [72]; one cause of which may be that NFAT5 localization and expression are impaired by elevated levels of proinflammatory cytokine production of these patients [73].

The role of NFAT5 in maintaining cell homeostasis and proliferation is weakened or indeed impaired in diabetes.

### 4.2. Pathogenic Role of NFAT5 in Diabetes Complications

**Figure 3:**
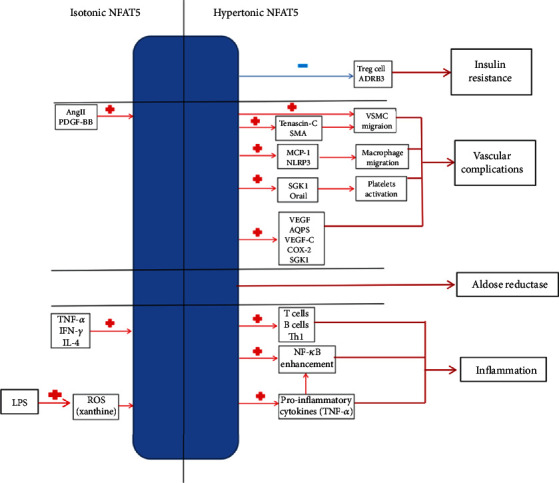
Pathogenic role of NFAT5 in diabetes complications (isotonic/hypertonic NFAT5). *β*3-Adrenoreceptor gene (ADRB3), angiotensin- (Ang-) II, aquaporin- (AQP-) 5, cyclooxygenase- (COX-) 2, interferon- (IFN-) *γ*, interleukin- (IL-) 4, lipopolysaccharide (LPS), monocyte chemoattractant protein- (MCP-) 1, nuclear factor- (NF-) *κ*B, nucleotide-binding oligomerization domain, leucine-rich repeat, and pyrin domain-containing protein 3 (NLRP3), platelet-derived growth factor- (PDGF-) BB, reactive oxygen species (ROS), regulatory (Treg) T cells, serum- and glucocorticoid-inducible kinase (SGK1), smooth muscle actin (SMA), tumor necrosis factor (TNF)-*α*, vascular endothelial growth factor (VEGF), and vascular smooth muscle cell (VSMC).

NFAT5 mediates pathologic responses. Upregulation of NFAT5 is observed during diabetic complications such as atherosclerosis [74], diabetic nephropathy [6], and retinopathy [75]. Clinically, the progression of diabetes complications may not stop even with glycemic control. NFAT5 can respond to other physiological and pathophysiological stimuli in tissues that do show large changes in tonicity, leading to the pathogenesis of diabetic complications. We summarize the pathogenic role of NFAT5 from four perspectives: AR, vascular complications, inflammation, and insulin resistance (Figure 3). These factors interact with each other and promote the development of diabetes complications.

#### 4.2.1. NFAT5 and AR

NFAT5 induces AR to catalyze the conversion of glucose to sorbitol to adapt to the hyperosmotic environment. The supply of glucose in patients with diabetes is higher than that in healthy people. AR is a key enzyme in the polyol pathway and critical factor in the pathogenesis of diabetic complications [76]. Rather than exerting a protective effect, upregulation of AR by NFAT5 predominantly aggravates diabetes. The transcriptional activity of NFAT5 enhances AR expression under conditions of hyperglycemia in cultured peripheral blood mononuclear cells and human mesangial cells, which were isolated from patients with diabetic nephropathy [77]. The DNA-binding activity of NFAT5 was found to be increased in peripheral blood mononuclear cells from patients with diabetes with nephropathy compared to that in an uncomplicated group without nephropathy [6, 77]. Elevated NFAT5 and AR protein levels were also observed in the retina of diabetic mice [78]. Additionally, NFAT5 upregulates retinal ganglion cell apoptosis in diabetic retinopathy. NFAT5 deficiency decreases AR expression and alleviates the retinopathy [75]. NFAT5 functions upstream of the polyol pathway to regulate the progression of diabetes.

#### 4.2.2. NFAT5 and Vascular Complications in Diabetes

Under hypertonic stress, NFAT5 directly upregulates the expression of tenascin-C and smooth muscle actin (SMA), which orchestrate the migration of vascular smooth muscle cell (VSMC) to promote maladaptive vascular remodeling processes such as arterial stiffening [79]. Angiotensin- (Ang-) II [80] and platelet-derived growth factor- (PDGF-) BB [81] are associated with prior cardiovascular events in diabetes and stimulate NFAT5 to regulate VSMC migration in the absence of hypertonic stress [82].

NFAT5 directly regulates monocyte chemoattractant protein (MCP)-1 [83] and nucleotide-binding oligomerization domain, leucine-rich repeat, and pyrin domain-containing protein 3 (NLRP3) [84], which induce macrophage migration and mediate endothelium innate immunity, resulting in the formation of atherosclerosis during the early stages of atherosclerosis. In a previous study, NFAT5 haploinsufficiency reduced atherosclerotic lesion formation by 73%. Transplantation of NFAT5+/-ApoE-/- marrow into NFAT5+/+ApoE-/- mice resulted in a similar 86% reduction in lesion formation [74]. NFAT5 haploinsufficiency also alleviated renal macrophage infiltration in a mouse model of diabetic nephropathy [7]. Hyperactivated platelets commonly detected in diabetes may contribute to cardiovascular complications [85]. NFAT5 upregulates serum- and glucocorticoid-inducible kinase (SGK1) and Orai1 expression in megakaryocytes, an effect that likely influences platelet sensitivity to stimulation [86].

Vascular endothelial growth factor (VEGF) and AQP5 promote the development of diabetic retinopathy, accompanied by retinal neovascularization and edema. Hyperosmotic expression of VEGF and AQP5 is stimulated by the activity of NFAT5 [87]. NFAT5 is an upstream regulator of vascular angiogenesis that induces VEGF-C [88], cyclooxygenase (COX)-2 [89], and SGK1 [90, 91], which stimulate atherosclerotic lesion development and aggravate vascular complications of diabetes. The expression level of NFAT5 was significantly higher in patients with type 1 diabetes mellitus (T1DM) and nephropathy compared to that in patients without microvascular complications [6]. NFAT5 knockdown in human umbilical vein endothelial cells (HUVEC) impeded angiogenic processes, which can also be found in mice with NFAT5 haploinsufficiency (NFAT5+/-) [92].

#### 4.2.3. NFAT5 and Inflammation in Diabetes

Persistent inflammation is an important inducer of the progression of diabetes complications. NFAT5 plays a predominant role in mediating inflammation and regulating immune responses. NFAT5 can be activated in leukocytes (T cells, B cells, and macrophages) under hypertonic stress [7]. NFAT5 upregulates the Th1-stimulatory cytokine interleukin 12 (IL-12) in classically activated macrophages and enhances the expression of the pro-Th1 mediators Fizz-1 and arginase 1. Heme oxygenase-1 (HO-1) [93], a stress-inducible protein, is induced by various oxidative and inflammatory signals, and its expression is regarded as an adaptive cellular response to inflammation and oxidative injury. NFAT5 suppresses the expression of HO-1 by blocking nuclear factor erythroid-2-related factor 2 (Nrf2) binding to the HO-1 promoter. Thus, NFAT5 enhances the functions of macrophages to promote Th1 (proinflammatory mode) polarization over Th2 (anti-inflammatory or regulatory mode) responses [94]. NF-*κ*B is regarded as a central mediator of inflammation and regulates various molecules involved in the early and various stages of the immune response in diabetes. NFAT5 binds to the TNF-*α* promoter without directly interacting with DNA; it shares the same binding site with NF-*κ*B [14]. NFAT5 is required for NF-*κ*B enhancement by recruiting transcriptional cofactor p300 through the p65-NFAT5-p300 complex through protein-protein interactions [95]. Other components of NF-*κ*B stimuli such as specificity protein 1 (Sp1) and RNA polymerase II also rely on NFAT5-dependent recruitment [95]. Additionally, NF-*κ*B does not respond to high glucose-related hyperosmotic stress [58]. NFAT5-sensitive proinflammatory cytokines such as TNF-*α* promote the activity of NF-*κ*B [96]. NFAT5 initiates the inflammatory response earlier than NF-*κ*B and plays a key role in activating NF-*κ*B at a very early stage.

Inflammation may still deteriorate under an isotonic state when blood glucose is well-controlled. NFAT5 can be activated by inflammatory signals such as TLR, TNF-*α*, IFN-*γ*, and interleukin-4 (IL-4) under isotonic conditions [12]. Neuroinflammation is also involved in this distinct regulatory pathway of NFAT5 [97]. Activation of NFAT5 under inflammation conditions accentuates the progress of inflammation and results in cell impairment. Lipopolysaccharide (LPS) and hypertonicity share NFAT5 as a core transcription factor, and these stimuli reciprocally inhibit the expression of downstream target genes [98]. Xanthine oxidase-induced reactive oxygen species (ROS), but not mitochondria-derived ROS, plays a key role in the progression of inflammation by activating NFAT5 [98, 99]. ROS function as molecular sensors to discriminate between lipopolysaccharide and osmotic stimuli in cells.

#### 4.2.4. NFAT5 and Insulin Resistance in Diabetes

Beige adipocytes are thermogenic adipocytes [100]. NFAT5 suppresses the process of white adipocyte turning to beige adipocyte, which results in insulin resistance [101]. Through DNA methylation, NFAT5 suppresses the expression of the *β*3-adrenoreceptor gene (ADRB3), which is a critical regulator of lipolysis and thermogenesis, thus increasing the risk of insulin resistance [101, 102]. NFAT5 haploinsufficiency attenuates adipogenesis and insulin resistance in mice with diabetes. The percentage of insulin-positive areas was lower in wild-type mice with diabetes mellitus than in NFAT5 haploinsufficiency mice with diabetes mellitus [103].

Regulatory T (Treg) cells play a fundamental role in the process of type 1 diabetes. It can keep autoreactive T cells “in check” and maintain immunological tolerance [104], thereby preventing the onset of islet autoimmunity and insulin resistance [105, 106]. miRNA181a increases the expression of NFAT5 in a tonicity-independent manner, which substantially inhibits Treg cell induction and thereby contributes to the development of insulin resistance [107].

## 5. Therapeutic Role of NFAT5 in Diabetes

**Figure 4:**
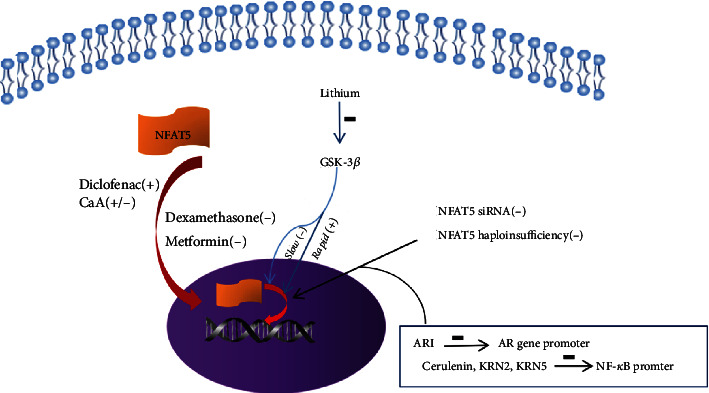
Gene and drug therapy of NFAT5 target for diabetes. Drugs (long red arrow) increase (+) or decrease (-) expression of NFAT5 mRNA and protein. Lithium inhibits phosphorylation of glycogen synthase kinase-3*β* (GSK-3*β*) and increases expression of NFAT5 rapidly, while long-term lithium exposure decreases NFAT5 expression. NFAT5 silencing RNA (siRNA) and haploinsufficiency disrupt the transcription of NFAT5. The DNA-binding activities of NFAT5 in the promoter of aldose reductase (AR) gene are significantly decreased in the presence of AR inhibitor (ARI). KRN2 and KRN5 derivate from berberine. Cerulenin, KRN2, and KRN5 selectively block NF-*κ*B binding to the NFAT5 promoter region and inhibit transcriptional activation of NFAT5 cyclosporin A (CsA).

Strategies for alleviating hyperosmotic stress and minimizing inflammation would be helpful for enabling NFAT5 to maintain its physiological function. Recent studies have identified NFAT5 as an effective target therapy for diabetes (Figure 4).

### 5.1. NFAT5 Gene Therapy

Suppression of NFAT5 expression is an effective therapeutic strategy for diabetic complications. In the presence of AR inhibitor (ARI), the DNA-binding activities of NFAT5 in the promoter of the AR gene were significantly decreased. The effect was more obvious in patients with diabetes experiencing nephropathy than in those without nephropathy [6]. After treatment with NFAT5 siRNA, the levels of AR and protein kinase C were decreased and cell proliferation and angiogenic processes were inhibited [75]. Additionally, the level of the apoptotic factor B cell lymphoma 2-associated X protein (Bax) was reduced and the survival factor B cell lymphoma 2 (Bcl2) was increased [78]. Downregulation of proapoptotic proteins and upregulation of antiapoptotic proteins were found to be induced by knockdown of NFAT5 in the retina of diabetic mice. Mice injected with NFAT5 siRNA showed reduced streptozotocin-induced diabetic retinopathy [78]. NFAT5^+/-^ mice maintained lower fasting glucose levels and improved glucose tolerance and insulin sensitivity compared to their wild-type littermates, both of which were fed a high-fat diet [103]. Compared with wild-type diabetic mice, diabetic NFAT5^+/-^ mice displayed decreased body weight, fat mass, hepatic steatosis, and macrophage infiltration. NFAT5 haploinsufficiency attenuates insulin resistance and suppresses diabetes-associated hepatic steatosis and neuroinflammation [104]. NFAT5 is a viable therapeutic target for blocking VSMC migration in occlusive vascular disease [79]. NFAT5 in bone marrow- (BM-) derived cells acts as a positive regulator that accelerates atherosclerotic lesion formation [74]. NFAT5 haploinsufficiency led to a significant reduction in aortic lesions in an in vivo model of atherosclerosis [56].

Transcriptional knockdown of NFAT5 treatment can be applied under conditions in which NFAT5 is overactive. This therapy alleviates autoimmune reactions in type 1 diabetes and could be partially extended to persistent inflammation in diabetic wounds or retinal neovascularization in the future. However, this treatment also suppresses the normal physiological activities of NFAT5, and thus, further studies on indication selection and course control are needed.

### 5.2. NFAT5 Drug Therapy

Lithium is widely used as a mood stabilizer to treat bipolar disorder. Patients with 5 years or more of lithium use show a significantly increased risk of nephrogenic diabetes insipidus [108]. Lithium has different effects on NFAT5 activity, depending on the environmental osmolality and exposure duration. Lithium inhibits phosphorylation of GSK-3*β*. Under isosmotic conditions, GSK-3*β* enhances the activity of the C-terminal transactivation domain of NFAT5, resulting in elevated expression of NFAT5 and its target gene HSP-70 [109]. Lithium can also suppress NFAT5 expression under hyperosmotic stress conditions. The effects of lithium depend on the protein abundance of NFAT5. High osmolality increases NFAT5 protein levels, whereas long-term lithium exposure decreases NFAT5, which subsequently decreases the expression of HSP-70 [106]. Studies on the relationship between NFAT5 and lithium can guide adjustments to the dose and course of treatment to reduce side effects. Lithium-mediated effects on NFAT5 activity other than those in the kidney require further analysis.

Topical administration of diclofenac [110] or cyclosporin A (CsA) [111] to the eyes induces NFAT5 expression and protects the ocular surface under hypertonic stress conditions. Patients with dry eye syndrome, which commonly occurs in diabetes, may benefit from NFAT5-targeted therapy. Apart from that, CsA prevents nuclear translocation of NFAT5 and inhibits osmotic response element-mediated reporter gene expression of kidney medulla cells and human hepatoblastoma cells [112]. CsA has an effect on NFAT5 kidney medulla cells, indicating that CsA nephrotoxicity may partially inhibit adaptive responses to hypertonicity of the urinary concentrating mechanism. It requires further study to confirm if the different effects of CsA are related to cell types.

Dexamethasone is effective for managing diabetic macular edema (DME), particularly for treating DME resistant to anti-VEGF therapy and in vitrectomized eye treatment of DME [113]. Dexamethasone also decreases NFAT5 mRNA expression under hyperosmotic stress conditions [114]. Few studies have examined the correlation between dexamethasone and NFAT5. Although anti-VEGF therapy is a first-line treatment for DME, there are some limitations because of the etiological factors of DME, which are varied and complex. Hyperosmotic stress influences neovascularization and edema development. NFAT5 regulates VEGF, AQP5, and COX-2 expression correlated with DME [87, 89, 115]. Therefore, NFAT5 shows potential as a new therapeutic target for DME.

Metformin, a well-known antidiabetic drug, inhibits the mRNA and protein expression of hypertonicity-induced NFAT5 and its downstream target genes [116].

Whether gene therapy or drug therapy, the key point consists of outbalancing the diverse functions of NFAT5, elevating physiological function, and minimizing pathologic effects.

NFAT5 is required for the molecular interaction between p300 and p65 [95]. This p65-NFAT5-p300 complex exerts unique effects, as increased NFAT5 expression leads to higher NF-*κ*B activity, resulting in the recruitment of additional p300 [95]. Thus, NF-*κ*B activity is increased by NFAT5. This effect is inhibited by cerulenin, an inhibitor of fatty acid synthesis. Cerulenin disrupts the p65-NFAT5-p300 complex without affecting the expression, DNA binding, and regulation of p65 [95, 117].

Berberine, a traditional Chinese herbal medicine, has shown antihyperglycemic activities and positive effects on diabetic complications [118]. High-throughput screening revealed that KRN2, 13-(2-flauoro)-benzylberberine, and its derivative KRN5 are derived from berberine, which can suppress NFAT5 expression specifically. By blocking NF-*κ*B binding to the NFAT5 promoter region, KRN2 and KRN5 selectively inhibits transcriptional activation of NFAT5 and downregulates the expression of proinflammatory NFAT5-target genes without preventing high-salt induction of NFAT5 and osmotic protection of the target genes of NFAT5 [119]. Berberine, a NFAT5-targeting therapy for diabetes complication patients, could inhibit the inflammatory effects without affecting its osmotic effects since the latter is involved in cellular homeostasis and cytoprotection.

For both gene and drug therapy, it is important to balance the diverse functions of NFAT5 to increase its physiological function while minimizing pathologic effects. ROS inhibitors are potential NFAT5-targeting therapeutic agents for patients with diabetes [98].

## 6. Future Directions and Conclusions

Although NFAT5 is identified as a central regulator of cellular osmoadaptive responses, the function and regulation of this protein are not well-understood. Emerging evidence has shown that its dysregulation results in or is associated with the pathogenesis of diabetes mellitus, which indicates NFAT5 could play a broader role far beyond osmoadaptation in a tissue-specific manner. Researches on the NFAT5 regulatory pathway are critical for certain clinical presentations and to explore novel therapeutic approaches. Although dozens of molecules and pathways have been identified to contribute to tonicity-dependent regulation of NFAT5 in vitro, it remains an open question that how do these work together to orchestrate a specific signal to NFAT5 in response to osmotic stress.

Recent studies have identified NFAT5 as an effective target therapy for diabetes. Traditional methods of reducing hypertonic exposure, such as glucose, are effective for diabetes treatment. Although several clinical trials have demonstrated the effective role of supplementation with NFAT5 target osmolytes for diabetes treatment, the potential benefit and long-term effects of these drugs are still uncertain. An ideal NFAT5-targeting therapy for diabetes should selectively inhibit inflammatory effects while maintaining the physiological function of NFAT5. Sensitive molecular sensors such as ROS look promising in counterbalancing the physiological role of NFAT5 in diabetes therapy. Studies on drug therapy of NFAT5 are limited. Extracts of traditional Chinese medicine may be an important direction of future research on NFAT5-targeted drugs. Therefore, gene regulator NFAT5 could be a potential therapeutic target for diabetes. Further studies on the crosstalk between NFAT5 and diabetes pathways will give new insights of treatments for diabetes.

## Figures and Tables

**Table 1:** Diabetes-associated intronic single-nucleotide polymorphisms in NFAT5 [6–10].

SNP	Allele	Position on Chr16	Trait
rs17297179	A	69607827	eGFR
rs1064825	G	69690317	eGFR
rs17297207	A	68200854	Persistent proteinuria (>0.5 g/day) >10 years with T1DM
rs862320	C	69651866	T2DM

Chr16: chromosome 16; eGFR: estimated glomerular filtration rate; SNP: single-nucleotide polymorphism; T1DM: type 1 diabetes mellitus; T2DM: type 2 diabetes mellitus.

**Table 2:** List of major kinases to regulate tonicity-dependent activation/inactivation of NFAT5.

Kinases	Upstream	Transactivating activity	Nuclear localization	Protein abundance
ATM [20–22]	c-Abl(+) P13K(+)	+	+	/
c-Abl [22]	/	+	+	/
CDK5 [23]	/	/	+	/
CK1 [24]	/	/	-	/
ERK1/2 [25, 26]	PKC*α*(+)	+	/	/
FAK [27]	/	/	/	+
Fyn [28]	/	+	/	/
GSK-3*β* [29]	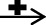 PKA(-) →P13K AKT1(-)	-	/	/
mTOR [30]	/	+	/	/
p38*α* [31–33]	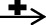 →MEKK3 MKK6(+) MKK3(+)	+	/	/
p38*δ* [31, 34]	MKK3(+)	-	/	/

ATM: ataxia telangiectasia mutated; c-Abl: cellular-abelsongene; CDK5: cyclin-dependent kinase 5; CK1: casein kinase 1; ERK1/2: extracellular signal-regulated kinase 1/2, FAK: focal adhesion kinase; GSK-3*β*: glycogen synthase kinase-3*β*; mTOR: mammalian target of rapamycin; +: increase; -: decrease; /: no effect or not mentioned.

## Abbreviations

AD: Transactivation domain
ADRB3: *β*3-Adrenoreceptor gene
AED: Auxiliary export domain
Ang-II: Angiotensin-II
AQP: Aquaporin
AR: Aldose reductase
ARI: Aldose reductase inhibitor
ATM: Ataxia telangiectasia mutated
Bax: B cell lymphoma 2-associated X protein
Bcl2: B cell lymphoma 2
BM-derived cells: Bone marrow-derived cells
BGT-1: Betaine GABA transporter-1
c-Abl: Cellular-abelsongene
Chr16: Chromosome 16
CDK5: Cyclin-dependent kinase 5
CK1: Casein kinase 1
COX-2: Cyclooxygenase-2
CsA: Cyclosporine A
DD: Dimerization domain
DME: Diabetic macular edema
eGFR: Estimated glomerular filtration rate
ERK1/2: Extracellular signal-regulated kinase 1/2
FAK: Focal adhesion kinase
GSK-3*β*: Glycogen synthase kinase-3*β*
HHS: Hyperglycemic hyperosmolar state
HO-1: Heme oxygenase-1
HUVEC: Human umbilical vein endothelial cells
HSP-70: Heat shock protein-70
IFN-*γ*: Interferon-*γ*
IL-4: Interleukin-4
IL-6: Interleukin-6
IL-12: Interleukin-12
iNOS: Inducible nitric oxide synthase
LPS: Lipopolysaccharide
LT-*β*: Lymphotoxin-*β*
MCP-1: Monocyte chemoattractant protein-1
mTOR: Mammalian target of rapamycin
NFAT5: Nuclear factor of activated T cells 5
NES: Leucine-rich canonical nuclear export sequence
NF-*κ*B: Nuclear factor-*κ*B
NLRP3: Nucleotide-binding oligomerization domain, leucine-rich repeat, and pyrin domain-containing protein 3
NLS: Nuclear localization signal
Nrf2: Nuclear factor erythroid-2-related factor 2
PDGF-BB: Platelet-derived growth factor-BB
RHD: Rel homology domain
ROS: Reactive oxygen species
SGK1: Serum- and glucocorticoid-inducible kinase
siRNA: Silencing RNA
SMA: Smooth muscle actin
SMIT: Sodium/myoinositol transporter
SNPs: Single-nucleotide polymorphisms
Sp1: Specificity protein 1
TauT: Taurine transporter
Thr135: Threonine 135
TLR: Toll-like receptor
TNF-*α*: Tumor necrosis factor-*α*
Treg cells: Regulatory T cells
Tyr143: Tyrosine 143
T1DM: Type 1 diabetes mellitus
T2DM: Type 2 diabetes mellitus
UT-A: Urea transporter
VSMCs: Vascular smooth muscle cell
VEGF: Vascular endothelial growth factor.
